# Supplementary material for: Integrated regulatory models for inference of subtype‐specific susceptibilities in glioblastoma
Source: Mol Syst Biol. 2020 Sep 25;16(9):e9506. doi: 10.15252/msb.20209506 (PMC7516378; doi:10.15252/msb.20209506)
Supplement: Supplementary file 1 — Expanded View Figures PDF [file MSB-16-e9506-s001.pdf]

Expanded View Figures

Figure EV1. Assessment of the quality of networks constructed by inTRINSiC.

A Proportion of edges in FANTOM5 brain-specific regulatory network covered by the DNaseI hypersensitivity site (DHS) network constructed from ENCODE cell line datasets using different PIQ score cutoffs.

B Alluvial diagram collating subtype label assignment of TCGA samples using classification scheme of Verhaak *et al* (2010) (left column) and that of Wang *et al* (2017) (right column).

C Area under precision-recall curve for the recovery of known interactions among transcription factors (TFs) by parameterized regulatory networks constructed with varying regularization strengths (connected orange dots). The same metrics were computed for networks built using linear regression for comparison (connected black dots).

D Histograms showing distribution of Pearson correlation coefficients between *F* value vectors obtained using TCGA subtype labels from Verhaak *et al* (Fig EV1B left column) and those obtained using re-assigned subtype labels from Wang *et al* (Fig EV1B right column). Mean of the distribution is significantly larger than 0 with estimated Wilcoxon test *P*-values of 2.13×10^{-86}, 8.02×10^{-86}, and 4.86×10^{-86} for Classical, Proneural, and Mesenchymal subtypes, respectively.

E Error rates of gene expression prediction for each glioblastoma multiforme (GBM) subtype using the final parameterized network. Left panel: symmetric mean absolute percentage error (sMAPE) was computed for each gene's expression values across all samples in a given subtype and the average sMAPE across all genes in each subtype is reported. Results did not significantly vary when median instead of mean sMAPE value across all genes were used (right panel). Bar plots show mean error rates in 5-fold cross-validation.

F Histograms of overlap (as quantified by the Jaccard index) between TF-target gene pairs inferred using the TCGA and CGGA datasets for up-regulation (left) and down-regulation (right) edges. Estimated Wilcoxon test *P*-values are 6.00×10^{-76} and 2.42×10^{-47} for the up- and down-regulation distributions, respectively.

G Subtype-specific plots showing a non-monotonic relationship between median TF regulatory capacity (calculated as median absolute value of $\log_2 F$ values for each TF in each subtype) and TF expression variability (calculated as coefficient of variation for gene expression).

© 2020 The Authors

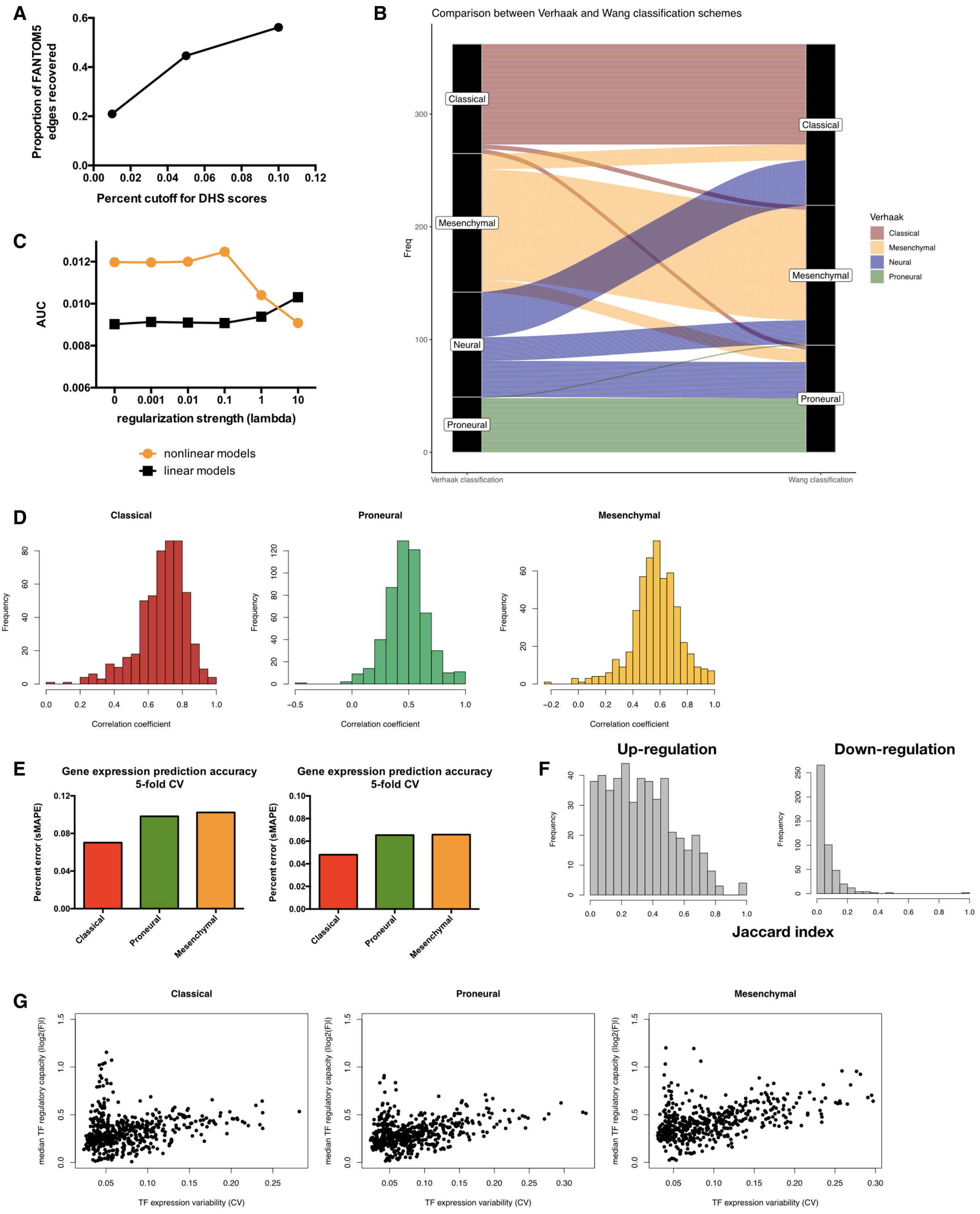

Figure EV1.

© 2020 The Authors

Figure EV2. GBM subtype-specific regulatory profiles built by inTRINSiC recover known biology, predict cell state plasticity and aid the inference of protein activities.

A Heatmaps of correlation coefficients among TF regulatory profiles in each GBM subtype. The Classical subtype heatmap was ordered using hierarchical clustering with the average linkage method as the reference and all other heatmaps were ordered in the same way for comparison. Only upper triangular matrices are shown.

B Histograms of Pearson correlation coefficients between transcription regulatory parameters inferred by inTRINSiC (*F* values) and ARACNe (mutual information). The RANSAC method was used to prevent correlation from being heavily skewed by extreme values.

C Overlap between TF-TF interactions inferred by inTRINSiC and ARACNe using correlation among pairs of TF regulatory profiles. Fisher's exact test *P*-values are 2.55×10^{-58}, 2.31×10^{-131}, and 4.63×10^{-95} for overlap between Classical, Proneural, and Mesenchymal datasets, respectively.

D Enrichment of known BIOGRID interactions recovered by inTRINSiC (dotted curve), ARACNe (solid curve), and GENIE3 (used in the SCENIC pipeline) plotted against increasingly relaxed threshold on TF-TF parameter correlation thresholds. *P*-value for Fisher's exact test of proportion of known interaction pairs among top 1% correlated TFs compared with selecting all possible interaction pairs: 1.655×10^{-12} for inTRINSiC, 1.19×10^{-17} for ARACNe, and 3.313×10^{-12} for GENIE3.

E UMAP projections of TCGA samples upon simulated knockdown of HOXA1 (left panel) and NEUROG1 (right panel). Each arrow points from the TCGA sample before perturbation to the same sample after perturbation in the same UMAP embedding.

F Number of proteins covered by VIPER and that covered by the exponential ranking protein activity inference function in inTRINSiC.

G, H Inferred response of STAT3 (i) and KRAS (j) protein activity to EGFR inhibition in each of the three subtypes predicted by VIPER (upper panels) or inTRINSiC (lower panels). *P*-values are calculated using Wilcoxon matched-pairs signed rank test. ****$P < 0.0001$, **$P < 0.01$. Bars represent mean $\pm$ SEM. # of patients in each subtype: Classical—139, Proneural—103, Mesenchymal—165.

© 2020 The Authors

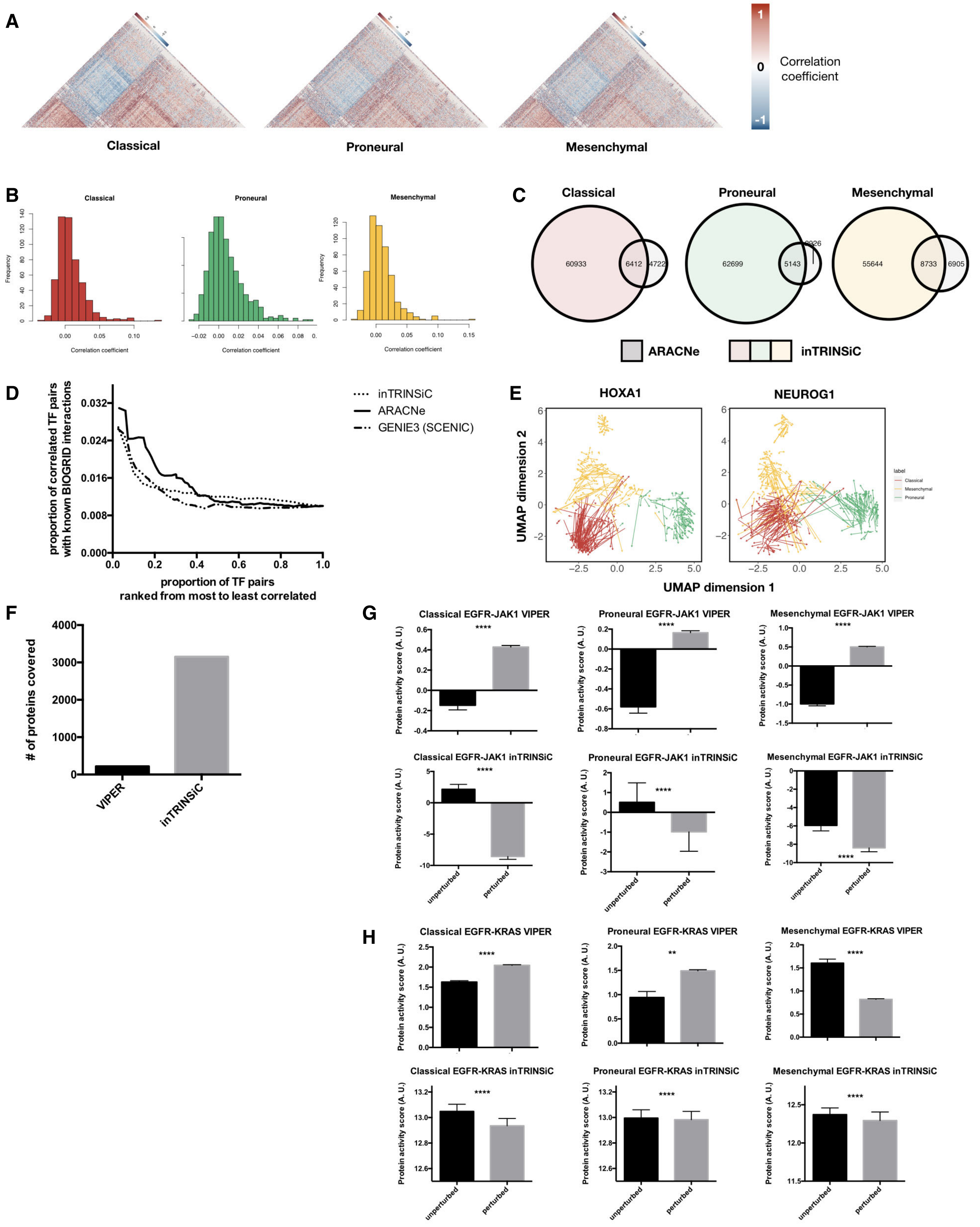

Figure EV2.

 © 2020 The Authors

Figure EV3. CCLE brain tumor cell lines co-cluster with TCGA patient samples in the transcriptome signature subspace.

A Heatmap of combined TCGA tumor sample and CCLE brain tumor cell line gene expression values (with top 2,000 median absolute deviation across all samples), ordered using hierarchical clustering with the complete linkage method. Top color codes above the first row indicate whether the column is a tumor sample (teal) or a cell line (magenta), and bottom color codes indicate the subtype that the sample is assigned. Prior to clustering, the TCGA and CCLE expression datasets were normalized using the RemoveBatchEffects function in the R limma package.

B t-stochastic neighbor embedding (t-SNE) plot of TCGA and CCLE brain tumor/cell line samples (color-coded using subtype labels) with perplexity set to 10. Solid dots are TCGA tumor samples whereas empty circles with a cross are CCLE samples.

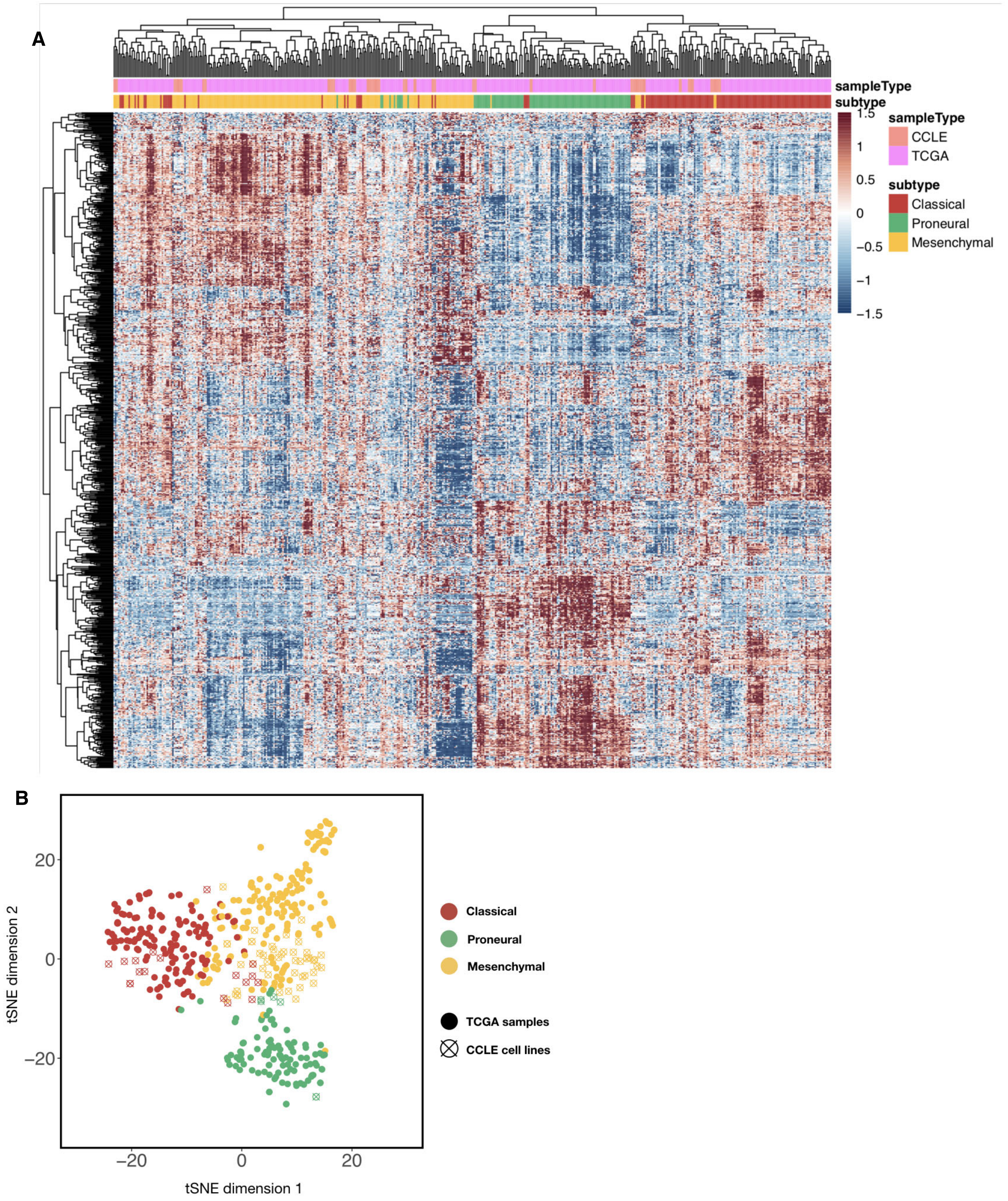

Figure EV3.

© 2020 The Authors

Figure EV4. Multilayer network information flow predicts changes in protein activity that in turn predicts subtype-specific gene essentiality in GBM.

A Heatmap of elastic net regression coefficients for predicting susceptibility to the perturbation of each TF. Shown are TFs with top absolute regression coefficients (maximum absolute value > 0.01 across all predictors), and rows and columns are both ordered using hierarchical clustering with the Weighted Pair Group Method with Centroid Averaging (WPGMC) method.

B WPGMC-clustered heatmap of correlation coefficients among the regression coefficient vectors of TFs.

C *In silico* knockdown of *NFE2* in TCGA GBM samples potentially confers a Proneural-specific therapeutic advantage. Shown are jitter plots of predicted essentiality scores of NFE2 in tumor samples grouped by subtype. Box plots show 25th, 50th, and 75th percentiles, and whiskers extend up to 90th and down to 10th percentiles. *P*-value for one-way analysis of means among scores across subtypes is 1.16×10^{-11} ($df1 = 2$, $df2 = 252.12$). # of patients in each subtype: Classical—139, Proneural—103, Mesenchymal—165.

D Kaplan–Meier curves for TCGA and CCGA Classical and Mesenchymal tumor patient survival, with patients segregated into MYBL2-high (salmon curve) and MYBL2-low (teal curve) groups. Log-rank test *P*-values are shown for each subtype. Number of patients in TCGA Classical subset: MYBL2-high—62, MYBL2-low—63, TCGA Mesenchymal subset: MYBL2-high—79, MYBL2-low—80. Number of patients in CGGA Classical subset: MYBL2-high—24, MYBL2-low—24, CGGA Mesenchymal subset: MYBL2-high—37, MYBL2-low—38.

© 2020 The Authors

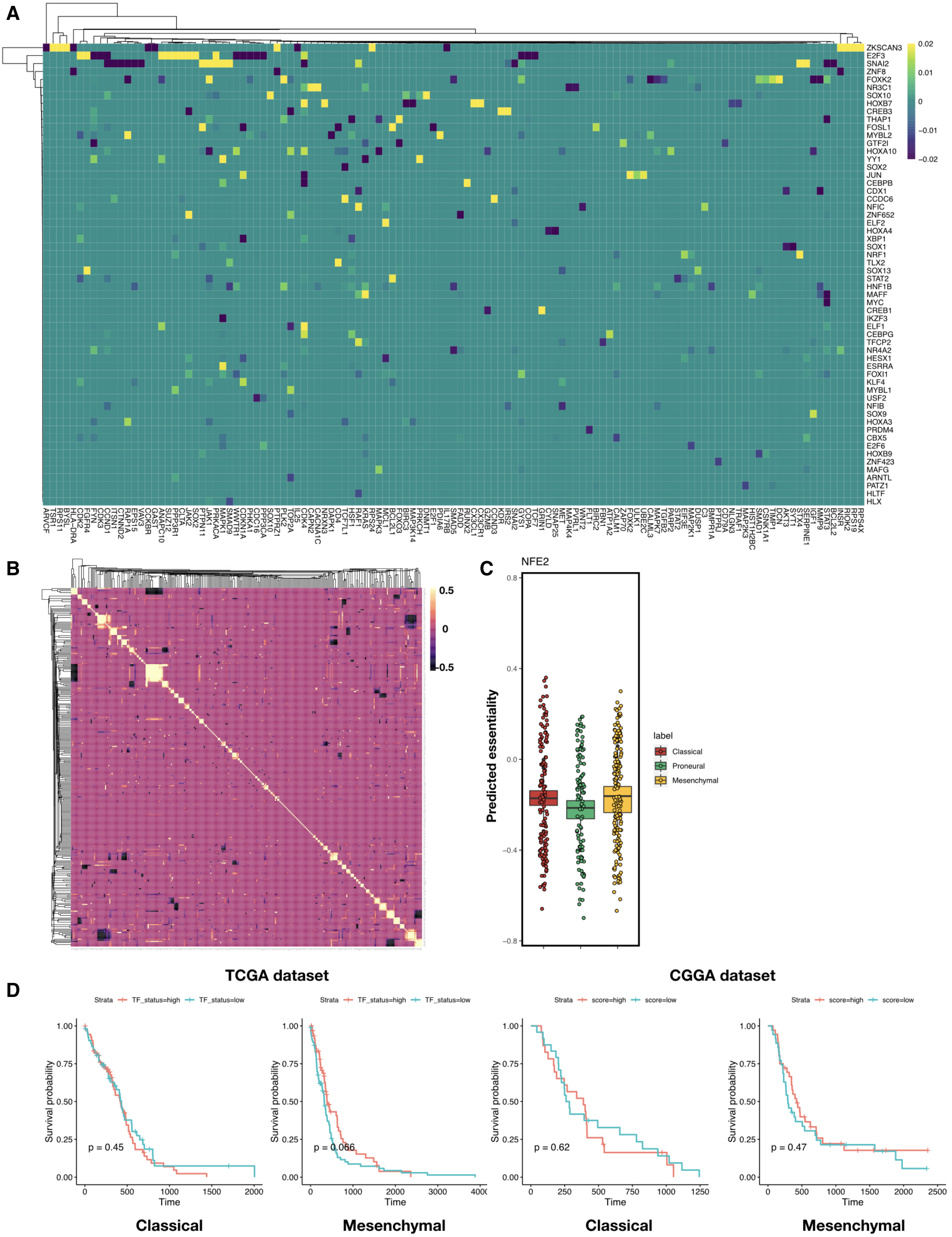

Figure EV4.

 © 2020 The Authors

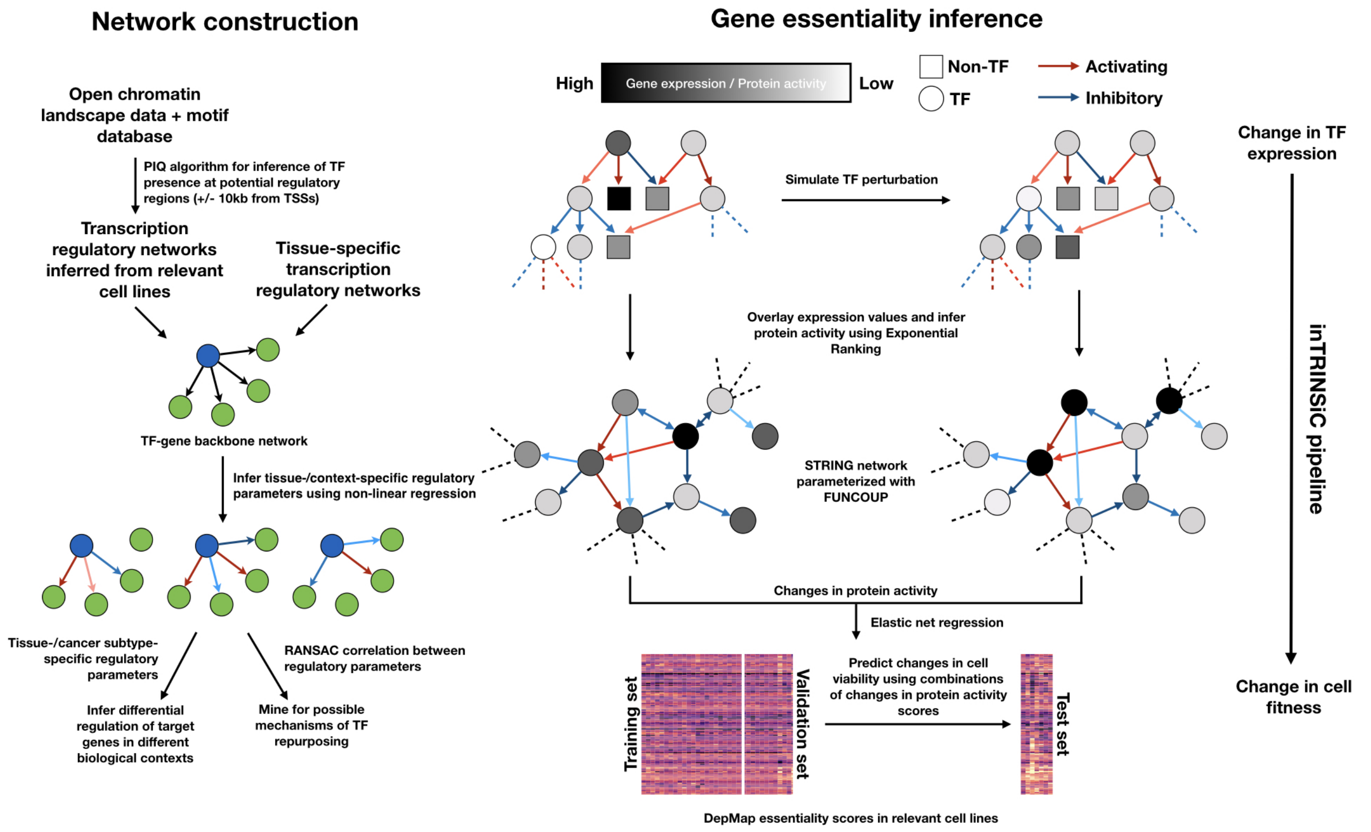

Figure EV5. A generalized inTRINSiC pipeline.

Schematic of the generalized inTRINSiC modeling framework. Left: procedures for constructing context-specific regulatory networks that readily accommodate additional mechanisms of transcription regulation. Right: using context-specific networks to perform *in silico* perturbation analysis and infer the relative importance of a given regulator in the network.

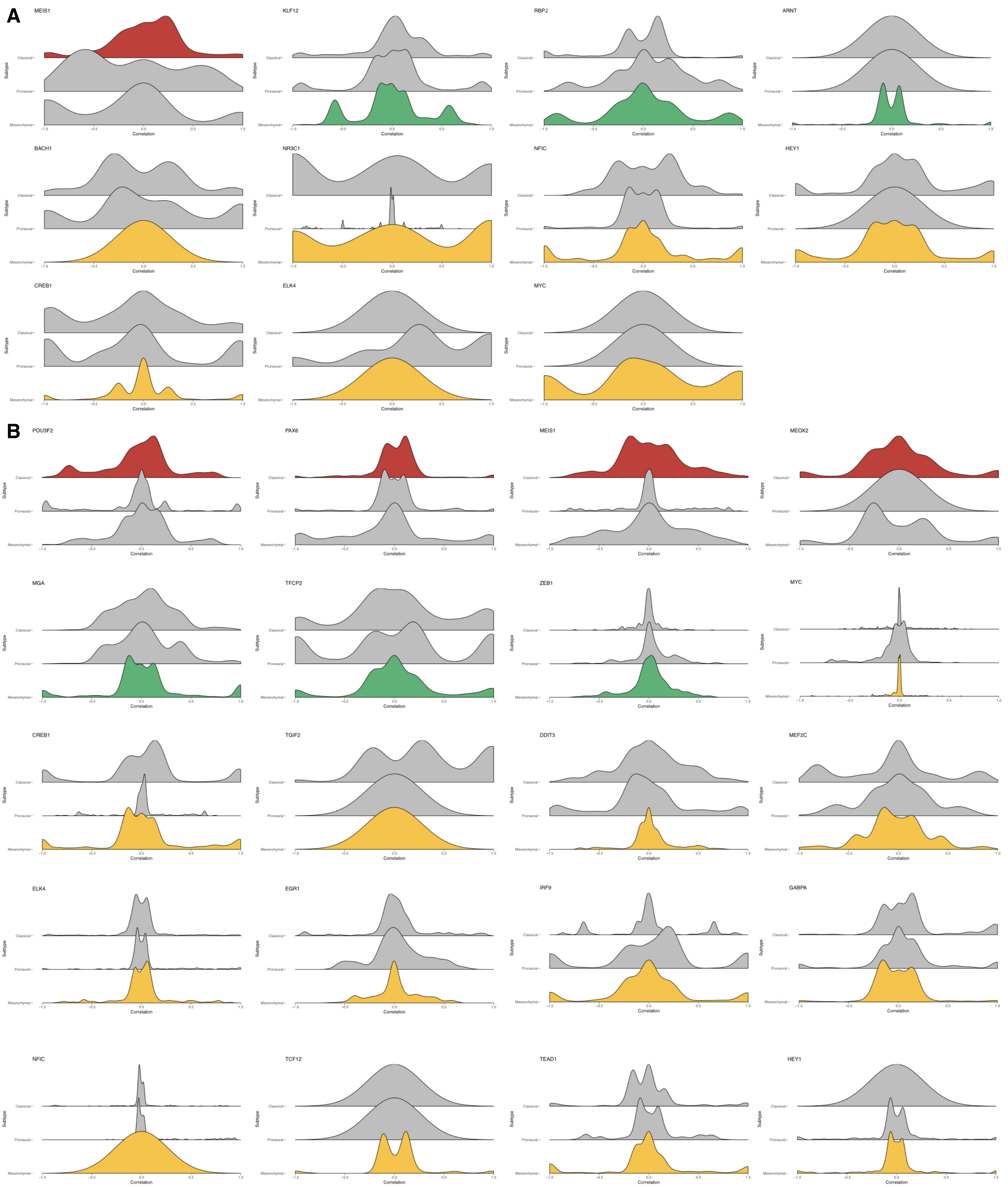

Figure EV6.

 © 2020 The Authors

◀

Figure EV6. Additional plots of TF partnering at differentially regulated genes in GBM.

A, B Histograms of correlation coefficients of signature TFs with other TFs at differentially regulated target genes where the $\log_2 F$ values at these target genes in the signature subtype are higher (A) or lower (B) than the average across all subtypes. Plots are grouped according to signature subtype. Histograms in each subplot are arranged in the order of Classical, Proneural, and Mesenchymal subtypes from top to bottom.

© 2020 The Authors
