# Supplementary material for: Integrated regulatory models for inference of subtype‐specific susceptibilities in glioblastoma
Source: Mol Syst Biol. 2020 Sep 25;16(9):e9506. doi: 10.15252/msb.20209506 (PMC7516378; doi:10.15252/msb.20209506)
Supplement: Supplementary file 2 — Table EV1 [file MSB-16-e9506-s002.docx]

| **Network Construction Method** | **inTRINSiC** | **ARACNe** | **SCENIC (GENIE3)** | **SYGNAL** |
| --- | --- | --- | --- | --- |
| Type of regulator-target relationship captured | Non-linear | Mutual information | Co-expression* | Co-expression |
| Pre-select regulator-target edges based on experimental evidence | Yes | No | No | Yes |
| Yields regulatory parameters with direct biological interpretations (magnitude and directionality etc.)? | Yes | No | No | No |
| Allows in silico perturbation of transcriptional regulators? | Yes | No | No | No |
| Allows explicit incorporation of additional mechanisms of transcription regulation (non-coding RNA, epigenetic etc.)? | Yes | No | No | Yes |

| **Protein Activity Inference Method** | **inTRINSiC** | **VIPER** |
| --- | --- | --- |
| Utilizes protein-protein signaling interactions | Yes | No |
| Independent of transcription regulation inference? | Yes | No** |

*: co-expression here is defined as a (linear or non-linear) relationship between the regulator and target genes inferred using random forest regression, where the relationship does not take any particular analytical form.

**: In an extension of the VIPER algorithm, residual post-translational signaling activities are inferred by regressing out transcriptional variance. However, the VIPER algorithm still relies on differential expression to infer either direct or indirect regulatory effects on protein activity.

**Table EV1** Comparisons of inTRINSiC’s features with that of other regulatory inference methods
